# The application of organoids in colorectal diseases

**DOI:** 10.3389/fphar.2024.1412489

**Published:** 2024-06-25

**Authors:** Yanxin Liu, Dongxu Wang, Yanhong Luan, Boqiang Tao, Qirong Li, Qiang Feng, Hengzong Zhou, Jianfeng Mu, Jinhai Yu

**Affiliations:** ^1^ Department of Gastric and Colorectal Surgery, General Surgery Center, The First Hospital of Jilin University, Changchun, China; ^2^ Laboratory Animal Center, College of Animal Science, Jilin University, Changchun, China; ^3^ Department of Oral and Maxillofacial Surgery, Hospital of Stomatology, Jilin University, Changchun, China

**Keywords:** colorectal cancer, organoids, tumor microenvironment, inflammatory bowel disease, gene editing

## Abstract

Intestinal organoids are a three-dimensional cell culture model derived from colon or pluripotent stem cells. Intestinal organoids constructed *in vitro* strongly mimic the colon epithelium in cell composition, tissue architecture, and specific functions, replicating the colon epithelium in an *in vitro* culture environment. As an emerging biomedical technology, organoid technology has unique advantages over traditional two-dimensional culture in preserving parental gene expression and mutation, cell function, and biological characteristics. It has shown great potential in the research and treatment of colorectal diseases. Organoid technology has been widely applied in research on colorectal topics, including intestinal tumors, inflammatory bowel disease, infectious diarrhea, and intestinal injury regeneration. This review focuses on the application of organoid technology in colorectal diseases, including the basic principles and preparation methods of organoids, and explores the pathogenesis of and personalized treatment plans for various colorectal diseases to provide a valuable reference for organoid technology development and application.

## 1 Introduction

“Colorectal diseases” is a collective term for benign and malignant diseases that occur within the colon, including colorectal cancer (CRC), inflammatory bowel disease (IBD), and intestinal infections. CRC is one of the most common colorectal diseases and may be related to changes in diet, increased obesity, environmental factors, and aging (Lu et al., 2021). CRC is the third most common cancer worldwide, accounting for 9.8% of all malignant tumors, and the second leading cause of cancer-related deaths, with a mortality rate of 9.2% (Sung et al., 2021). The World Health Organization has estimated the occurrence of 2.2 million new CRC cases and 1.1 million CRC-related deaths annually worldwide by 2030 (Ferlay et al., 2019).

IBD is a non-specific inflammatory disease of the digestive tract. It encompasses Crohn’s disease (CD) and ulcerative colitis (UC) and is characterized by chronic inflammation leading to mucosal damage in the digestive tract (Chen et al., 2021). In 2017, the total number of patients with IBD worldwide reached 6.8 million (Ng et al., 2017), making this disease a massive burden on global public health services. The primary medical treatments for IBD include aminosalicylates, corticosteroids, biological agents, and immunosuppressants (Cai et al., 2021). Surgical intervention is required when medical treatment is ineffective or serious complications arise (Lamb et al., 2019). While biological agents can change the course of IBD, about one-third of patients fail to respond to them (Zhang et al., 2019). This indicates that existing treatment schemes have difficulty achieving satisfactory clinical efficacy.

Organoids are three-dimensional structures that self-assemble from stem cells, pluripotent cells, or tissue-specific cell types under *in vitro* culture conditions and have highly similar structures and functions to the source tissue or organ (Lancaster and Knoblich, 2014). In 2009, Sato et al. (2009) used leucine-rich repeat-containing G protein-coupled receptor 5 (*Lgr5*)^+^ small intestinal stem cells to cultivate tissues with intestinal crypts and villi structures, creating a precedent for the organoid research field. This microenvironment biomimetic cell culture method has since been applied to more types of cell culture, and various patient-derived organoids (PDOs) have been successfully constructed (Barker et al., 2010; Sato et al., 2011; Rossi et al., 2018; Nikolaev et al., 2020).

PDOs can more faithfully simulate the biological behavior of tissues *in vivo* and have a more stable genome than traditional two-dimensional (2D) cell culture models. In addition, PDOs are easier to culture, with short construction times and high success rates, and can facilitate cell transfection and high-throughput screening more efficiently than patient-derived tumor xenograft (PDX) models (Table 1) (Bleijs et al., 2019; Tuveson and Clevers, 2019). Normal colon and tumor organoids can be cultured from specimens from patients with CRC with a success rate of over 90% (Van De Wetering et al., 2015). Even after long-term *in vitro* culture, organoids retain the original tumor tissue characteristics, gene expression profile, and metastatic potential *in vivo* (Jensen et al., 2023). Organoids effectively simulate the microenvironment of multiple cell interaction types in tissues *in vivo* and can reflect the tissues’ physiological and pathological conditions (Li and Izpisua Belmonte, 2019). Therefore, organoids are suggested as a source of new ideas and methods for diagnosis, drug screening and development, and gene therapy for colorectal diseases.

**TABLE 1:** Comparison of preclinical models in cancer research.

	2D cell lines	PDXs	Organoids
Establishment success rate	High	Relatively low	Relatively high
Maintenance	Easy	Difficult	Easy
Gene editing	Able	Unable	Able
Cost	Low	High	Relatively high
Expansion	Quick	Slow	Quick
Reproducibility	High	Moderate	Low
Representativeness	Low	Low	High
Tumor immune microenvironment	Unable to recapitulate	Partial recapitulation	Partial recapitulation
Tumor heterogeneity	Unable to recapitulate	Retain	Retain
Complexity	Low	High	High

## 2 Application of organoids in CRC

Molecular genetic studies have shown that CRC is a highly heterogeneous tumor that arises from various genetic variants via two major pathways: chromosomal instability and microsatellite instability (MSI) (Li et al., 2021). Different molecular subtypes of CRC have different drug sensitivities. However, a few patients are resistant to first-line CRC treatments. Therefore, individualized precision therapy is the key to treating advanced CRC (Keum and Giovannucci, 2019).

Previous *in vitro* studies on CRC mainly used 2D tumor cell lines and PDX models (Mouradov et al., 2014; Inoue et al., 2019). The 2D model can neither simulate tumors’ spatial structure and heterogeneity *in vivo* nor reflect the cell interactions in the CRC microenvironment. While PDX models can strongly simulate the structure, heterogeneity, and physiological environment of tumors, the immune-deficient mice used to create them lack normal immune function and cannot be used for tumor immunity-related research and drug development (Neto et al., 2023). Unlike traditional models, organoid models derived from cancer tissues retain the molecular and biological characteristics of the malignant tissues (Mao et al., 2023). The organoid model provides a unique platform for studying tumors’ biological characteristics, mechanisms of development and progression, drug sensitivity, and personalized therapy based on the mutated genes.

### 2.1 Organoid models for CRC

**FIGURE 1:**
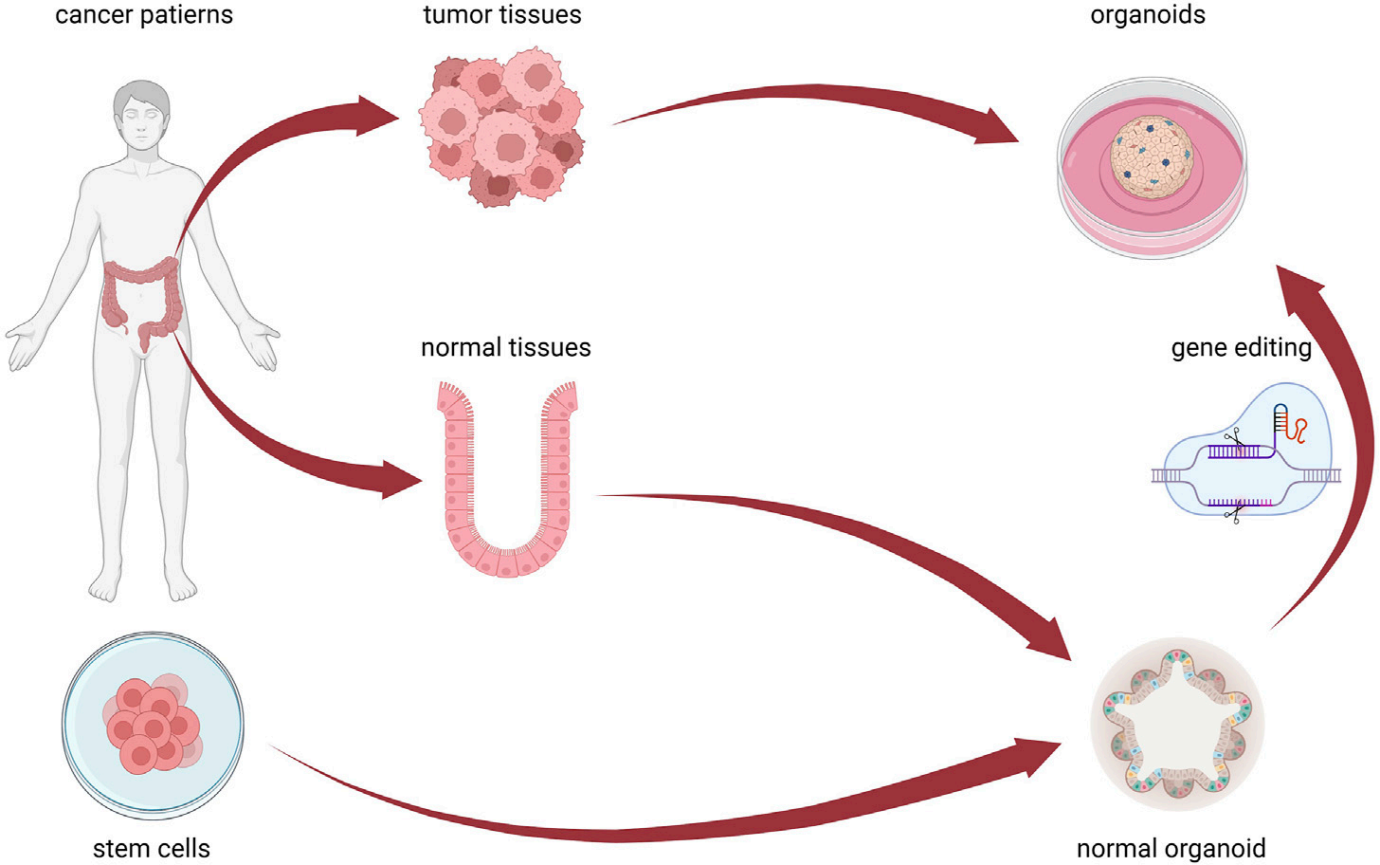
CRC organoid cultivation method. Patient-derived tumor-like organoids can be isolated and cultured from cancer tissue. Normal intestinal organoids can be isolated and + cultured from normal tissues, induced by stem cells to differentiate, and transformed into cancerous organoids through gene editing technology. Figure 1 was created with BioRender.com (accessed on April 4th, 2024).

CRC organoids are generally constructed from tumor specimens or biopsies obtained by surgical resection (Figure 1), with a success rate of 60%–90% (Dijkstra et al., 2018). After enzyme treatment, the tissue can be embedded in Matrigel for suspension culture or cultured at a gas-liquid interface. To construct CRC organoids, epidermal growth factor (EGF), Wnt pathway agonist R-spondin 1 (RSPO1), and bone morphogenetic protein inhibitor noggin (NOG) must be added to replace the missing AChE-related signaling molecules. In addition, nicotinamide, transforming growth factor-beta (TGF-β) inhibitor a83-01, p38 mitogen-activated protein kinase (MAPK) inhibitor sb202190, and prostaglandin E2 can make the culture conditions suitable for the long-term growth of human primary colorectal adenocarcinoma (Van De Wetering et al., 2015). Organoid construction efficiency is reported to be improved by adjusting the medium composition and oxygen concentration (Fujii et al., 2018).

In addition to using patient tumor tissue samples, CRC organoids can be constructed using gene editing technology to introduce specific gene mutations or modifications into stem cell-derived organoids to simulate genetic variation in CRC (Figure 1). APC Wnt signaling pathway regulator (*Apc*), transformation-related protein p53 (*Trp53*/*Tp53*), Kirsten rat sarcoma viral oncogene homolog (*Kras*), and SMAD family member 4 (*Smad4*) gene mutations were successfully introduced into the colon organoids of wild-type mice via short hairpin RNAs, biologically transforming them into invasive adenocarcinoma (Li et al., 2014). Specific mutated genes were introduced into normal colon organoids using clustered regularly interspaced short palindromic repeats (CRISPR)-CRISPR-associated protein 9 (Cas9) gene editing technology, revealing the role of EGF, Wnt, and TGF-β signaling pathways in the malignant transformation of the colon epithelium (Roper et al., 2017). After mutating *KRAS*, *APC*, *TP53*, and *SMAD4*, human intestinal organoids could grow in culture without the presence of stem cell niche molecules, and the p53 stabilizer nutlin-3 allowed them to exhibit the biological characteristics observed in invasive tumor tissues *in vivo* (Drost et al., 2015).

Utilizing organoids as preclinical models revealed the functional implications of sequential oncogenic mutations for promoting tumor proliferation, migration, and metastatic colonization in CRC (Fumagalli et al., 2017). The CRC development process was simulated by introducing specific mutations into human stem cell-derived organoids using CRISPR technology, uncovering the underlying mechanisms and providing crucial insights for developing related treatment methods (Drost et al., 2017). These studies improve the understanding of heterogeneous tumor genotypes and are of great significance for their personalized treatment (Schütte et al., 2017).

### 2.2 Application of organoids in the tumor microenvironment

The tumor microenvironment (TME) is a dynamic space within and around the tumor that largely determines its heterogeneity and plasticity (Almusawi et al., 2021). The conditions under which these microenvironments are reproduced in organoids are crucial for studying CRC. Tumor organoids typically consist of cancer cells derived from epithelial tissues, and establishing a TME in these organoids relies on artificial construction methods. Some CRC types, such as high MSI (MSI-H), B-Raf proto-oncogene, serine/threonine kinase (*BRAF*) mutant, and mucinous adenocarcinoma, depend highly on the TME, making it difficult to construct organoids successfully (Li et al., 2020). Therefore, the advanced coculturing of tumor-associated fibroblasts, immune cells, and other TME components with organoids is attractive.

Cancer-associated fibroblasts (CAFs) are a major TME component. They secrete stimulatory signals to support tumor development, suppress immunity, and promote drug resistance. Coculturing CRC-PDOs with two CAF types demonstrated that inflammatory CAFs promoted the epithelial-mesenchymal transition of CRC, while tumor-associated myofibroblasts reversed this effect (Mosa et al., 2020). Coculturing patient-derived CAFs with CRC-PDOs revealed that the tumor proliferated continuously without the addition of common growth factors to the PDO culture. Moreover, gene expression profiling and enrichment analysis of the model with TME components found that the immune response-related pathways missing in the non-coculture model were reactivated, showing that introducing TME components improved the simulation of the model (Luo et al., 2021).

Organoids play a crucial role in epigenetics. Preclinical studies of organoid and xenograft models have shown that DCAF1-mediated EZH2 phosphorylation plays an important role in gene reactivation in CRC cells (Ghate et al., 2023). The study of organoids in CRC also revealed that the absence of H4K20me3 mediated by Suv4-20h2 promotes the development of right CRC tumors through chromatin compaction (Boonsanay et al., 2023). In addition, organoid research uncovered the role of key molecular mediators. For example, SOX9 promotes stem cell activity and hinders normal differentiation in the development of CRC (Liang et al., 2022). Nuclear TYRO3 receptor tyrosine kinase molecular mediators such as BRD3 and MMP-2 play important roles in the metastatic process of CRC (Hsu et al., 2023).

The recent emergence of immunotherapy has advanced tumor-specific immunological *in vitro* models for patients with cancer. A personalized organoid platform for patients was constructed by coculturing organoids with homologous peripheral blood lymphocytes, and tumor-responsive T-cell were successfully enriched from the peripheral blood of MSI-H-type patients, confirming their cytotoxicity against homologous PDOs (Dijkstra et al., 2018). This finding enables the dynamic evaluation of individualized immune therapy efficacy for patients under minimally invasive conditions and offers the potential to utilize peripheral blood for adoptive T-cell therapy.

Chimeric antigen receptor (CAR) cell therapy holds great promise for microsatellite-stable CRC with weaker immunogenicity. In 2019, Schnalzger et al. (2019) reported a coculture platform of CRC organoids with CAR-NK cells, which allowed for the dynamic and quantitative monitoring of CAR-mediated cytotoxicity. Their findings suggested that the targeted effect of CAR cells on tumor-specific antigens enabled them to express tumor antigen-specific cytotoxicity even with trace amounts of tumor antigen expression or in complex microenvironments.

Furthermore, a previous study reported an air-liquid interface method for culturing mechanically dissected tumor tissue (Neal et al., 2018). This method constructed an organoid-like model containing the intrinsic tumor stroma, allowing the coculturing of tumor cells with naturally embedded immune cells. The tumor-infiltrating lymphocytes (TILs) within the model accurately retained the T-cell receptor repertoire of the original tumor. This organoid-like model simulated anti-programmed cell death 1 (PDCD1/PD-1) immunotherapy and exhibited tumor antigen-specific TIL activation and cytotoxic responses consistent with those *in vivo*. Moreover, this model successfully simulated the intrinsic immune components of the TME rather than peripheral blood immune cells, which are related to immune checkpoint inhibitors.

Organoids can reproduce the heterogeneity and microenvironment of tumors, laying the foundation for high-throughput drug sensitivity screening, personalized precision therapy, and further research on immunotherapy.

### 2.3 Application of organoids in anti-tumor drug screening

In recent years, new therapeutics, such as targeted and immunotherapeutic drugs, have improved patients’ prognoses. However, due to tumor heterogeneity and the close relationship between patients’ individual differences and drug efficacy, some patients still do not benefit from existing treatments (S et al., 2022). In addition, there is a gap between the current commonly used 2D culture tumor cell model and the cell characteristics of the original tumor, making it difficult to identify suitable drugs for personalized medication and new drug development (Hay et al., 2014). The organoid model has advantages in predicting the sensitivity of anti-tumor drugs over the classic 2D method of drug screening with CRC cell lines (Castro et al., 2021). Tumor cells within the same tumor can have different genetic and phenotypic characteristics, making treatment effective on some tumor cells but not others (Burrell et al., 2013). Compared to 2D tumor cell lines and PDX models, organoids can effectively simulate the TME *in vivo* and form a basis for high-throughput drug screening (Horvath et al., 2016).

In 2015, Van De Wetering et al. (2015) reported the first successful application of PDOs for high-throughput drug screening for CRC. They screened 83 drugs using a CRC organoid library from 20 patients and found that RAS-mutant organoids were insensitive to EGF receptor (EGFR) inhibitors. Vlachogiannis et al. (2018) reported the successful construction of a PDO model library using metastatic CRC samples from 16 patients. With this model, the therapeutic efficacies of regorafenib and cetuximab could be predicted with a sensitivity of 100% and a specificity of 93%. Yao et al. (2020) cultured 80 CRC organoids and assessed their sensitivity to radiotherapy and chemotherapy. Varinelli et al. (2024) cultured 12 CRC peritoneal metastases-derived organoids and demonstrated their utility in evaluating treatment plans at the patient level. These studies successfully connected cancer genetics with clinical trials, addressed the limitations of cell line-based and PDX models, and demonstrated that CRC organoids could be *in vitro* models for screening drugs and advancing precision medicine.

Schütte et al. (2017) tested the efficacy of 16 clinical drugs on organs derived from patients with CRC. They found that 14 genes, including regulator of G protein signaling 4 (*RGS4*), brain abundant membrane attached signal protein 1 (*BASP1*), and insulin-like growth factor 2 (*IGF2*), were associated with resistance to EGFR inhibitors and could serve as markers of insensitivity to EGFR inhibitors. The simultaneous blockade of the KRAS signaling pathway overcame resistance to targeted therapy of the MAPK pathway in metastatic CRC (Verissimo et al., 2016). In addition, organoid technology could be used to culture normal colorectal tissue from patients’ tumors, screen anti-tumor drugs, reduce damage to normal cells, and reduce toxic and side effects (Vlachogiannis et al., 2018). These results suggest the potential value of using PDO models for the preclinical evaluation of anticancer inhibitors.

Organoid chip technology could be used for drug screening. An organoid chip is a microfluidic system that can reproduce three-dimensional (3D) structures and cell-cell and cell-material interactions within tissues *in vitro* (Low et al., 2021). Combining tumor organoid microfluidic chips and various cell sensors enables the monitoring of tumor cell status. Organoid chips could be used as an efficient, high-throughput tumor drug screening platform (Sun et al., 2019). Carvalho et al. (2019) developed a CRC microfluidic chip and successfully constructed a vascular support network with human colon microvascular endothelial cells, successfully simulating the correlation between drug concentration gradient and therapeutic effectiveness. Wang et al. (2020) pioneered a non-contact model system combined with digital sensing technology to non-invasively monitor CRC organoid proliferation and metabolism. Aleman and Skardal (2019) constructed a multi-site metastatic tumor chip covering CRC, liver, lung, and endothelial cells and other organoids that were interconnected by circulating fluid and used fluorescence imaging technology for cell tracking. The above-mentioned studies provide more efficient models for advancing and evaluating anticancer medications.

**FIGURE 2:**
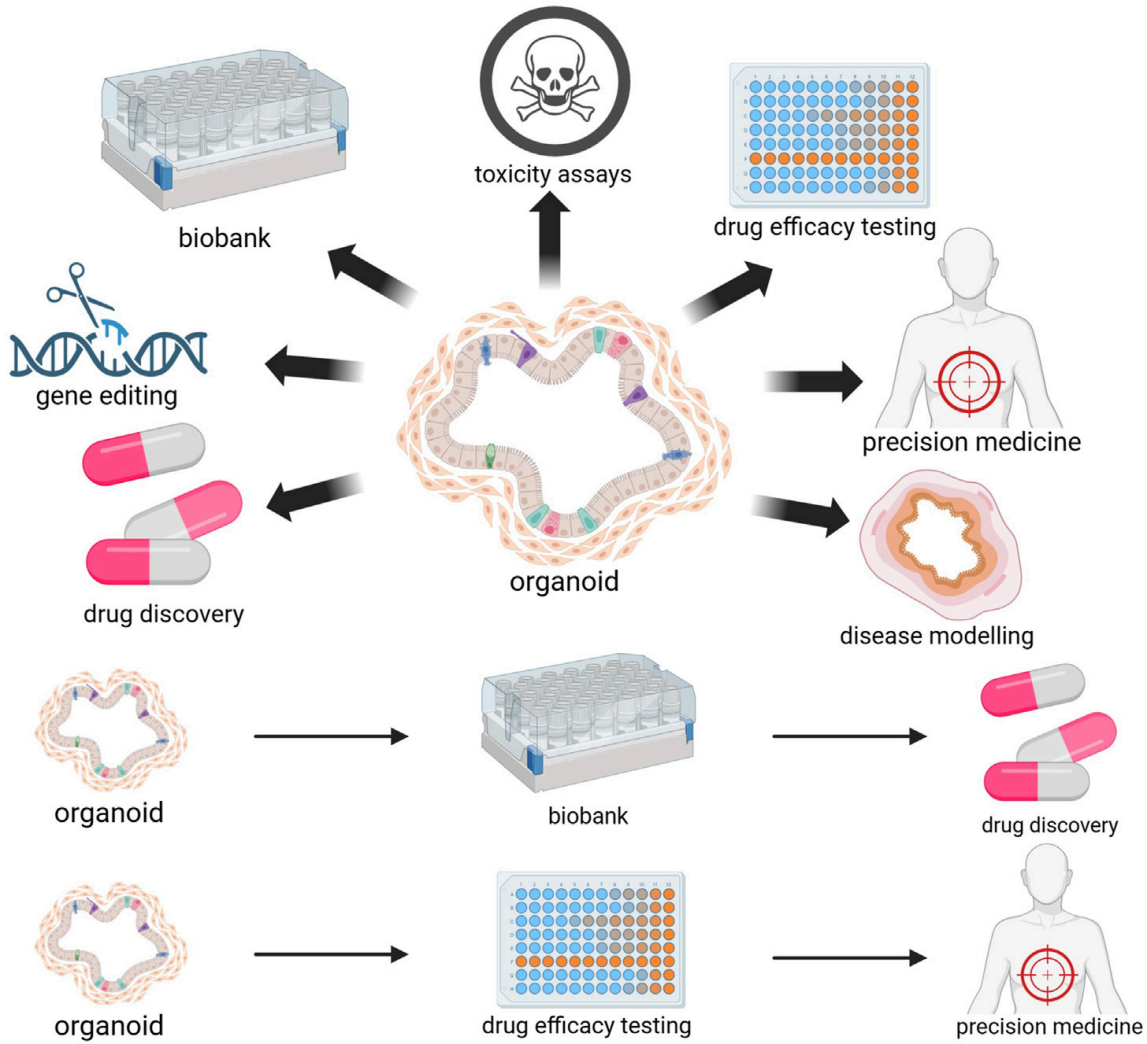
Biomedical applications of organoids. The utilization of tumor organoids derived from patients enables the prediction of individualized drug responses and personalized treatment outcomes. The freezing of organoids enables the establishment of organoid biobanks. Organoids are an ideal model for drug screening and toxicity testing and can be used for drug development. Organoids can also serve as disease models and undergo gene editing to promote research on CRC pathogenesis and physiological pathology. Figure 2 was created with BioRender.com (accessed on April 4th, 2024).

The application of tumor organoids has shown great potential in overcoming the challenges caused by tumor heterogeneity and individual differences. Organoids have shown significant value in predicting drug reactions and enabling high-throughput drug screening, providing a valuable platform for efficient drug application in CRC (Figure 2).

### 2.4 Application of organoids in personalized medicine

Personalized medicine aims to tailor treatment options for patients according to their specific genomics and metabolomics (Hamilton et al., 2021). The organoid model is close to the patient’s physiological microenvironment, allowing for a more precise estimation of their drug reactions (Li et al., 2022). A prospective clinical study (Ooft et al., 2019) investigated the application of PDOs of CRC metastatic tumors to identify patients who failed to respond to standard chemotherapy regimens. Its results showed that PDOs accurately predicted the clinical responses of patients receiving irinotecan treatment. PDOs can provide accurate response prediction and guidance for personalized treatment. Ganesh et al. (2019) collected tumor tissues from patients with CRC at different stages and performed organoid culture. They also analyzed the clinical response of each patient to clinical chemotherapy or radiotherapy. They found a strong correlation between the projected outcomes from organoids and the clinical response observed in patients after treatment.

A prediction model was developed to analyze the radiotherapy response data of patients using a machine-learning algorithm (Park et al., 2021). When applied to 33 patients diagnosed with rectal cancer, the prediction accuracy of the radiosensitivity model of patient-derived tumor organoids was above 89%.

Tailored treatment plans could also be developed for patients by studying the impact of different treatment plans on organoids. Schwank et al. (2013) successfully applied CRISPR-Cas9 technology to human-derived organoids. They used this technology to repair the F508del mutation in the CF transmembrane conductance regulator (*CFTR*) gene commonly found in patients, reinstating CFTR function in intestinal organoids. Geurts et al. (2020) also successfully corrected *CFTR* mutations using CRISPR-based technology and achieved the functional restoration of CFTR in intestinal organoids. Indeed, organoid technology can help treat CRC tumors by testing single or combination treatment plans in PDO models to determine the most effective plan for each patient and achieve personalized treatment (Figure 2).

### 2.5 Organoid biobanks

Organoid biobanks can provide data for drug development and contribute more to personalized and regenerative medicine than PDX models (Gunti et al., 2021). Van De Wetering et al. (2015) established the first CRC organoid biobank in 2015, significantly promoting genomic and functional research on organoids at the patient level. Another tumor tissue organoid biobank was created using 55 patients with CRC, shedding light on the functional connections and differences between tumors’ genetic variation, ecological requirements, and biological phenotypes (Fujii et al., 2016). Another biobank of colorectal organoids derived from samples from 41 patients, including normal colon organoids derived from adjacent normal tissues, was established with an approximately 77% success rate (Ganesh et al., 2019). A biobank consisting of 80 colorectal tumor organoids was also successfully constructed, demonstrating the accurate prediction of neoadjuvant radiotherapy and chemotherapy efficacy for locally advanced CRC (Yao et al., 2020). An organoid biobank offers a collection of cancer organoid cultures encompassing the intricacies of diverse tumor subtypes (Zhou et al., 2021). This significant advancement greatly facilitates progress in novel drug development and screening processes (Figure 2).

## 3 Application of organoids in IBD

**FIGURE 3:**
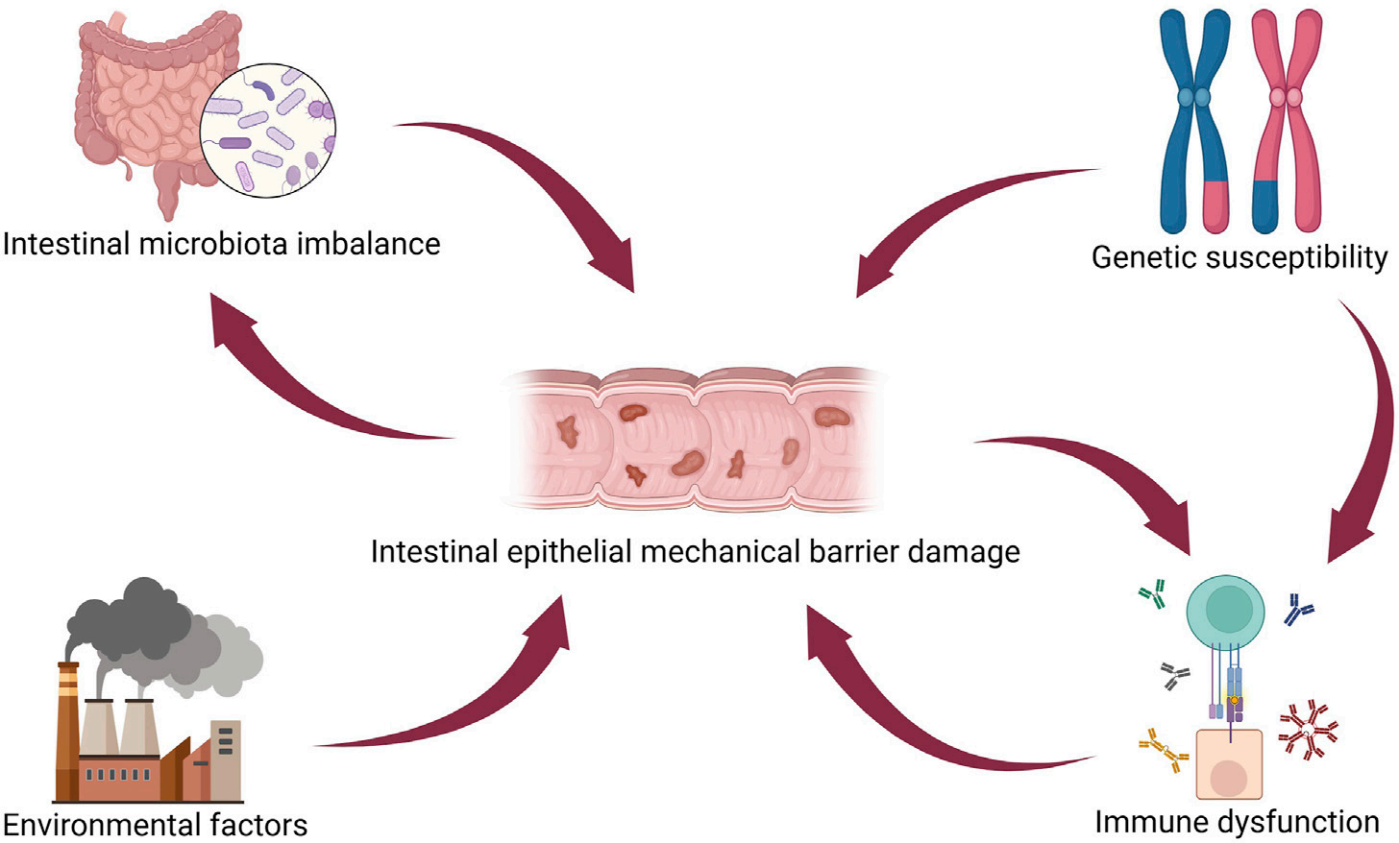
IBD pathogenesis. Intestinal epithelial injury directly interacts with several other pathogenetic mechanisms in IBD, such as intestinal microbiota imbalance, genetic susceptibility, environmental factors, and immune dysfunction. Figure 3 was created with BioRender.com (accessed on April 4th, 2024).

IBD pathogenesis is related to genetic predisposition, immune dysfunction, intestinal epithelial mechanical barrier damage, intestinal microbiota imbalance, and stimulation by environmental factors (Le Berre et al., 2023). As one pathogenic mechanism of IBD, intestinal epithelial mechanical barrier damage is interrelated with several other pathogenic factors and contributes significantly to IBD pathogenesis (Figure 3) (Khare et al., 2019). A genomic association study on IBD development identified some genes, such as innate immunity activator (*INAVA*/*C1orf106*), ring finger protein 186 (*RNF186*), and hepatocyte nuclear factor 4 alpha (*HNF4A*) as associated explicitly with maintaining epithelial barrier integrity (Graham and Xavier, 2020). Impairment of the intestinal epithelial barrier is a pivotal element of IBD pathogenesis. However, colorectal epithelial cell lines cannot fully recapitulate the heterogeneity of the intestine (Onozato et al., 2020). Moreover, their ability to reproduce the pathological and physiological characteristics of IBD is limited (Kotla and Rochev, 2023). Animal models of IBD are expensive, have long cultivation cycles, and cannot recapitulate human physiological characteristics (Schulte et al., 2019). Therefore, there is a pressing need for novel *in vitro* models to advance the understanding of IBD’s pathogenesis and develop more effective treatment strategies.

The composition and arrangement of intestinal organoid cells are similar to the structure of intestinal epithelium, and they can be continuously passaged and cultured *in vitro* (Sato et al., 2009). Their chromosome number and expression profile are also highly consistent with those of their source (Dotti et al., 2017). Cultivating intestinal organoids requires a small amount of tissue, and both endoscopic biopsy and surgical specimens can be used as tissue sources. Moreover, the mucosa of both inflammatory and non-inflammatory sites in the intestine can form intestinal organoids *in vitro* (Liu et al., 2023). These results indicate that gut-like organs hold great promise as reliable instruments for studying IBD pathogenesis and drug screening.

### 3.1 Construction of organoid models for IBD

Intestinal organoid culture medium primarily consists of extracellular matrix (ECM) gel and culture medium. The ECM gel mainly provides mechanical support while transmitting molecular signals required for cell growth (Rezakhani et al., 2021). The culture medium is categorized into expansion and differentiation media. The expansion medium contains many growth factors essential for the proliferation of colonic stem cells and is used for the long-term cultivation and amplification of colonic organoids (Almeqdadi et al., 2019). The differentiation medium is based on the expansion medium but lacks components such as Wnt family member 3A (WNT3A). It is used to induce the transformation of stem cells into diverse terminally differentiated colonic epithelial cell lineages (Flood et al., 2023).

There are currently two well-established methods for constructing intestinal organoids: one involves using adult stem cells (ASCs) (Wallach and Bayrer, 2017) and the other entails directing the differentiation of induced pluripotent stem cells (iPSCs) into intestinal organoids (Tsuruta et al., 2020). Essentially, they recreate the *in vitro* microenvironment of the colon epithelium, facilitating the proliferation and specialization of cells found in the colon, resulting in the development of organoid structures comprised of a comprehensive range of colonic epithelial cell lineages (Zachos et al., 2016).

The construction of colonic organoids based on ASCs involves initially collecting colonic tissue specimens through surgical or endoscopic procedures, followed by the subsequent isolation and purification of colonic LGR5^+^ stem cells. Next, these cells are combined with an ECM and multiple crucial growth factors, which support the continuation of the ASCs’ ability to self-renew and undergo differentiation (Sato et al., 2011). The construction of colonic organoids from iPSCs necessitates emulating embryonic gut development *in vitro*. First, iPSCs are induced to differentiate into a definitive endoderm layer. This is then directed to differentiate into posterior endoderm and subsequently expanded to produce colonic organoids (Múnera et al., 2017).

Dotti et al. (2017) successfully constructed a UC organoid model by culturing colon tissue samples from patients with UC *in vitro*. They found that it maintained genetic and biological characteristics highly consistent with those of the body tissue during long-term culture and passage. Colonic organoids constructed *in vitro* are highly similar to colonic epithelial tissue regarding cellular composition, tissue architecture, and specific functions (Zachos et al., 2016). Colonic organoids can effectively recapitulate the characteristics of colonic epithelial tissue in an *in vitro* culture environment.

### 3.2 Application of organoids in IBD pathogenesis

With their pluripotency, gene specificity, and structural polarity, intestinal organoids are progressively becoming a cellular experimental platform for IBD. Screening crucial pathways and transcriptional mechanisms driving epithelial dysfunction in patients with IBD within intestinal organoids is pivotal in elucidating IBD pathogenesis (Roh et al., 2019). Ishibashi et al. (2018) demonstrated that crypt stem cells derived from the diseased mucosa of patients with UC exhibited long-term differential transcriptional characteristics within organoids. They found that the antimicrobial peptide C-type lysozyme (*LYZ*), aquaporin 8 (*ACP8*), and transmembrane mucin 12 (*MUC12*) were downregulated, potentially leading to functional defects in colonic mucosal epithelial cells and contributing to the persistence of UC.

Sarvestani et al. (2018) performed immunohistochemical and next-generation sequencing analyses on an organoid model of UC constructed *in vitro*. Their findings revealed a remarkable level of agreement between the genomic and proteomic characteristics of the organism and this particular model. Single-cell sequencing of CD-derived intestinal organoids showed a notable disruption in the expression of *LYZ*, an antimicrobial peptide. Furthermore, there were distinct variations in the expression of markers associated with stem cells (Suzuki et al., 2018).

Hammoudi et al. (2022) found that mucosal T-cell originating from the same individual could directly trigger the death of epithelial cells. There was a direct association between T-cell infiltration in organ samples and epithelial cell death, which could be inhibited by blocking the lymphocyte-epithelial cell interaction through integrin subunit alpha E (ITGAE/CD103) and killer cell lectin-like receptor K1 (KLRK1/NKG2D) blocking antibodies.

Rees et al. (2020) discovered that the endoplasmic reticulum stress pathway is dysregulated in colonic organoids derived from both UC and CD. This dysregulation may increase the functionality of Toll-like receptor 5 (TLR5), elevating the secretion of interleukin 8 (IL8) and persistently activating peripheral dendritic cells, ultimately resulting in mucosal autoinflammatory responses.

### 3.3 Application of organoids in IBD treatment

IBD treatment mainly relies on drugs. Intestinal organoids can comprehensively simulate the internal environment and are highly similar in structure and function to the intestinal epithelium; thus, they can be used for drug detection. The clinical conversion success rate of IBD drugs in the gut organoid model has significantly increased (Kopper et al., 2021). Organoid models allow for evaluating the efficacy of conventional drugs and investigating novel drugs for treating IBD (Lacombe et al., 2022). Corticosteroids are commonly used to treat IBD (Pithadia and Jain, 2011). Using a confocal microscope, researchers detected the presence of fluorescein isothiocyanate-dextran 4 (FD4) infiltrating the lumens of intestinal organoids (Xu et al., 2021). Further research found that treating IBD organoids with corticosteroid prednisolone significantly reduced FD4 infiltration in the lumens and reduced the expression of inflammatory factors, indicating that corticosteroids are effective in treating IBD.

Treating intestinal organoids with tumor necrosis factor (TNF)-α resulted in the internalization and abnormal degradation of E-cadherin and decreased tight junction protein 2 (TJP2) levels (Khare et al., 2019). Treatment with 5-aminosalicylic acid (5-ASA) or azathioprine (AZTP) restored E-cadherin and TJP2 levels on the cell membrane to normal. These findings confirmed the ability of AZTP and 5-ASA to treat IBD, which is consistent with previous clinical research results (Swidsinski et al., 2007).

Lee et al. (2021) found in a study of 3D patient-derived intestinal organoids from CD patients that the reconstruction rate and cell viability were significantly impaired following TNF-α exposure. Kawamoto et al. (2019) examined the impact of infliximab, an anti-TNF-α medication, on intestinal organoids. They found that cotreatment of organoids with infliximab and TNF-α did not significantly affect their vitality or morphology but notably reduced ubiquitin D (*UBD*) expression, suggesting that infliximab has anti-inflammatory effects in treating IBD. Lloyd et al. (2020) added the macrolide antibiotic clarithromycin to colon organoids from healthy individuals. They found that clarithromycin had antibacterial effects and inhibited intestinal dermatitis.

In addition to medication, autologous intestinal transplantation after the *in vitro* expansion of self-derived intestinal organoids is a promising treatment approach for intractable ulcers and other disease manifestations in patients with IBD. Compared to conventional drug therapy, organoid mucosal therapy modulates the stem cell microenvironment at the lesion sites and promotes ulcer healing (Okamoto et al., 2020). Yui et al. (2012) found that colonic organoids cultured *in vitro* and then transplanted into a mouse model with acute UC induced by dextran sodium sulfate (DSS) could precisely reach the affected colonic epithelium and effectively restore the damaged tissue. They confirmed that expanding colonic stem cells *in vitro* and reintroducing them into the body could promote colonic epithelial regeneration and cure colonic mucosal damage. Watanabe et al. (2022) demonstrated that colonic organoids transplanted via the rectum into mice with UC could effectively reach and repair the damaged intestinal epithelium. Fordham et al. (2013) transplanted mouse small intestinal organoids into a mouse colonic injury model and found that they differentiated into colonic-like epithelial tissue, indicating that intestinal organoids possess immature cells that can adapt to the transplantation site by altering their phenotype. In a separate study by Sugimoto et al. (2018), normal human colonic organoids were successfully transplanted into the colons of immunodeficient mice, where they retained the characteristics of human colonic tissue and remained viable. These findings suggest that human intestinal organoid transplantation holds tremendous potential for treating IBD.

## 4 Application of organoids in intestinal infection

With improved sanitation conditions and advances in medical care, there has been a significant decrease in outbreaks of gastrointestinal infectious diseases and a noticeable reduction in mortality rates associated with infectious diarrhea (Meisenheimer Es et al., 2022). Intestinal organoid culture systems faithfully recapitulate the environmental conditions of the intestinal epithelium, allowing us to further explore the complex intestinal microbial ecosystem (Hentschel et al., 2021).

### 4.1 Construction of intestinal infection organoid models

**FIGURE 4:**
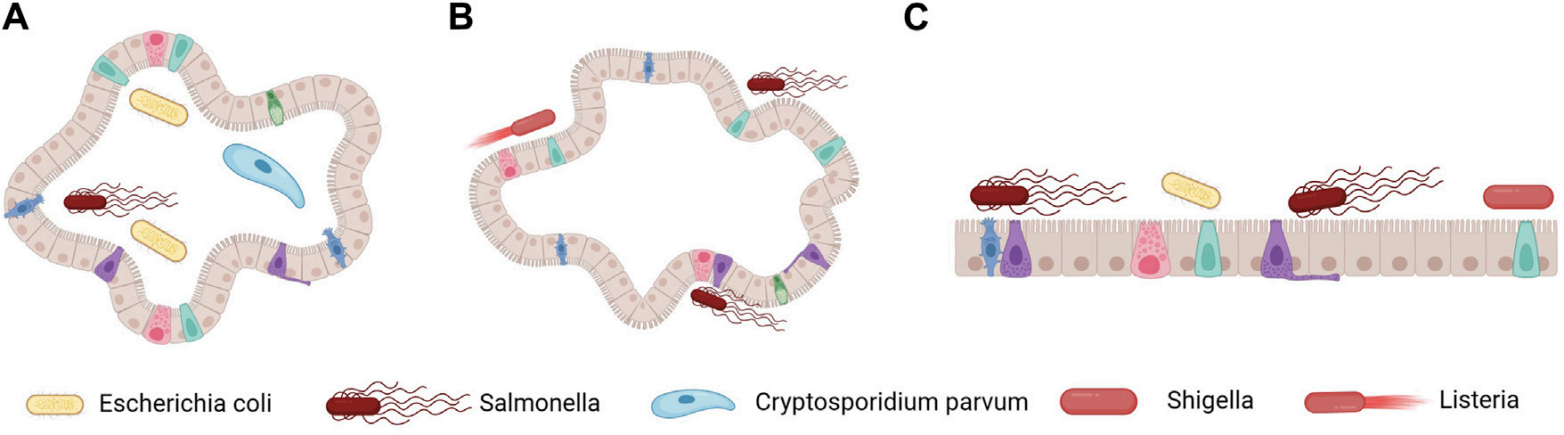
Methods for constructing organoid models of intestinal infection. **(A)** Microorganisms are introduced into the cavity of 3D organoids via microinjection, thereby achieving microbial epithelial interaction at the top. **(B)** The polarity of intestinal compounds is reversed, and microorganisms are added to the external medium. **(C)** Organoids are enzymatically dissociated to form 2D polarized monolayers, and microorganisms are added at the top or outer base. Figure 4 was created with BioRender.com (accessed on April 4th, 2024).

By co-cultivating intestinal organoids with pathogens, it is possible to generate organoids that simulate intestinal infections. These organoids have an inward-facing layer of epithelial cells, requiring the microinjection of microorganisms into the organoid lumen. This method has been successfully used to construct various organoid models of intestinal infection, including ones for enterohemorrhagic *Escherichia coli* (Pradhan and Weiss, 2020), *Salmonella enterica* serovar Typhi (Geiser et al., 2021), and *Cryptosporidium parvum* (Heo et al., 2018) infections (Figure 4). Moreover, organoids can sustain the growth of microbial communities from human fecal isolates within the cavity. However, the workload required for the intraluminal microinjection of organoids is high, making it difficult to control the diversity of infections (Williamson et al., 2018).

An inverted, apical-out organoid culture model has been developed to address these challenges. The methods for its construction involve removing the ECM and culturing the organoids in low-adherent plates, resulting in polarity inversion while maintaining functional and barrier integrity (Figure 4) (Co et al., 2019; Co et al., 2021). However, these methods result in the inability to access the basolateral side and still do not address the issue of infection diversity. An alternative strategy that overcomes these limitations involves culturing individual cells derived from organoids in a single layer (Roodsant et al., 2020). This method entails enzymatically dissociating the organoids into single cells, which are then seeded onto transwell inserts, ensuring the retention of the complexity of intestinal epithelial cells while establishing proper polarity and barrier formation. This approach enables access to both the apical and basal sides of the intestinal epithelium.

The single-layer model derived from human intestinal organoids can recapitulate the different intestinal cell populations, making it a valuable tool for studying specific infections in the gut (Figure 4). This method has been successfully used to study infections caused by pathogens such as *Shigella*, *Salmonella*, and pathogenic *E. coli* (Vandussen et al., 2015; Nickerson et al., 2021). Organoid technology can also be used to study viral invasion and intracellular replication. Notably, in the case of previously uncultivable viral agents such as norovirus (Ettayebi et al., 2016), human intestinal organoids have been crucial in constructing *in vitro* models. These models have been improved to investigate the interactions between viruses and intestinal cells and the host response to viral infections.

### 4.2 Application of organoids in intestinal infection

Gut organoid models can reveal the infection mechanism of intestinal pathogens. Saxena et al. (2016) demonstrated that the human rotavirus RV1 vaccine strain induced the expansion of small intestinal organoids through the action of the enterotoxin nonstructural protein 4 (NSP4) fragment, leading to a diarrheal response resembling physiological conditions. The rotavirus evades the host’s innate immune response by interfering with the nuclear factor kappa B (NF-κB) pathway in epithelial cells through nonstructural proteins 1 (NSP1) and 3 (NSP3), inhibiting the secretion of type III interferons after the activation of pathogen-associated molecular patterns (Saxena et al., 2017). Compared to traditional cell lines, such as the MA104 strain of rhesus monkey embryonic kidney cells and the CV-1 strain of African green monkey kidney cells, rotavirus demonstrates enhanced replication capacity in intestinal organoids.

Ettayebi et al. (2016) demonstrated that norovirus can utilize histo-blood group antigen receptors in the gastrointestinal tissue to exert its invasive effects when exogenous bile is added to the organoids. Drummond et al. (2017) observed that the specific cytotoxic effects of enterovirus type 11 could disrupt crypt structures, mislocalize tight junction proteins, and induce the release of inflammatory mediators. In contrast, no antiviral response was detected in intestinal organoids infected with coxsackievirus B and enterovirus type 71.

Intestinal organoids have also revealed the segment-specific and cell-specific characteristics of pathogen infections. For example, common pathogens causing childhood diarrhea, such as enteropathogenic *E. coli*, exhibit a stronger affinity for duodenum, ileum, and colon organoids (Holly and Smith, 2018). Additionally, various pathogens infect different cell types during the invasion process. Rotavirus primarily invades enterocytes and enteroendocrine cells (Saxena et al., 2016). Enterovirus type 11 cannot replicate in goblet cells, while enteric adenovirus type 5p preferentially infects goblet cells (Rajan et al., 2018). Using 3D confocal reconstruction techniques, Park et al. (2016) observed the entire process of *S*. *enterica* serovar Typhi-induced epithelial microvillus fold formation, intracellular bacterial replication, and necrotic cell shedding.

## 5 Application of organoids in regenerative medicine

The amount of tissue required for organoid cultivation is minimal, and most tissues can be obtained through minimally invasive procedures (Yi et al., 2021). The culture environment of organoids closely resembles human tissue and allows for large-scale *in vitro* cultivation, making organoid transplantation feasible (Bock et al., 2021). Tissue-specific ASCs within organoids can differentiate into relevant tissues or organs, enabling the direct induction of tissue regeneration during transplantation and maximizing the effectiveness of regenerative treatment (Choi et al., 2023). Organoids derived from ASCs exhibit a reduced likelihood of tumor development. Moreover, their direct transplantation at the injury site reduces their potential migration and dissemination to other organs. Using autologous cells in organoid therapy also decreases the likelihood of immune tolerance. Finally, most indications for organoid-based therapy can be treated with minimally invasive transplantation guided by endoscopy or ultrasound (Li et al., 2019).

Sugimoto et al. (2018) demonstrated that the transplantation of colon organoids into the inflamed intestines of mice induced by DSS and human-derived intestinal organoids into immunodeficient mice with mucosal damage both led to intact epithelial function and lineage tracing, confirming the excellent transplantability and high plasticity of the colon epithelium. Animal-derived decellularized ECM was found to be a suitable culture matrix for supporting organoids and *in vivo* transplantation, with modified organoid culture efficiency approaching that of matrix gel (Giobbe et al., 2019). Meran et al. (2020) developed allogeneic intestinal scaffolds, where donor-derived organoids were transplanted onto decellularized scaffolds to form repopulated grafts. These grafts maintained their luminal structures long after transplantation into mice. Okamoto et al. (2023) used a tissue-engineering approach with embryonic and iPSCs to generate human intestinal organoids, which can be combined with neural cells to gain intrinsic motility function.

Bioengineering studies on the vascular structures associated with organoids have also made it possible to transplant large tissue blocks (Grebenyuk and Ranga, 2019). Brassard et al. (2021) proposed creating a small intestine by combining 3D bioprinting technology with organoids. Using 3D bioprinting of human umbilical vein endothelial cells, mesenchymal stem cells, and intestinal organoids, they systematically fabricated centimeter-scale tubular intestinal epithelial tissue with a glandular-villus-like structure, connective tissue, and a vascular network. Sugimoto et al. (2021) constructed functional small intestine-like colons using ileum-derived organoids, achieving the reconstruction of the entire small intestine by replacing colon epithelial tissue and providing a feasible strategy for treating short bowel syndrome. In a non-clinical study, Jee et al. (2021) found that colon organoid-based regenerative therapy effectively treated radiation proctitis and restored damaged colon epithelial structure and integrity. Therefore, intestinal organoids have opened up a new avenue for research in colonic regenerative medicine.

## 6 New technologies for organoid construction

### 6.1 Applications of CRISPR-Cas9 technology in organoids

Gene editing is an emerging molecular biology technique that involves artificially altering a specific gene locus to modify the expression characteristics of the target gene, facilitating the study of gene function (Huang et al., 2021). CRISPR-Cas9 is a novel gene editing technology that utilizes an RNA-mediated adaptive immune defense system first discovered in 1987 (Ishino et al., 1987). CRISPR-Cas9 mainly comprises the *Cas9* gene, an RNA-guided endonuclease, and the CRISPR sequence, which consists of multiple conserved repeat sequences and interval sequences with a regular pattern. The Cas9 nuclease can recognize and cleave complementary DNA strands specific to the target site under the guidance of the corresponding guide RNA (Doudna and Charpentier, 2014; Shalem et al., 2014; Manghwar et al., 2020).

CRISPR-Cas9 technology offers advantages over traditional gene editing techniques, such as high flexibility, cost-effectiveness, and ease of use, making it the most convenient and powerful tool for gene editing (Karimian et al., 2019). The advent of CRISPR-Cas9 technology significantly simplified the challenges associated with human gene editing, and it found wide applications in the organoid field. Gene modification techniques based on CRISPR-Cas9 can introduce arbitrary combinations of cancer gene alterations into normal organoids to design cancer organoid models (Tuveson and Clevers, 2019). Utilizing CRISPR-Cas9 technology to edit commonly mutated genes in CRC and introduce them into normal human colon organoids ultimately results in CRC models with different phenotypes (Matano et al., 2015). Fujii et al. (2015) simulated the early development and progression of CRC by combining organoids with CRISPR-Cas9 technology. Artegiani et al. (2020) described a CRISPR-Cas9-mediated homology-independent targeted integration technique in organoids that enabled the precise integration of exogenous DNA sequences into organoids. Fumagalli et al. (2017) simulated the evolution of colorectal adenoma-carcinoma *in vivo* by transplanting colon organoids containing different mutated genes, elucidating how gene alterations in the WNT, EGFR, TP53, and TGF-β signaling pathways contribute to the growth, migration, and metastasis of colorectal tumors. CRISPR-Cas9 provides an effective tool for studying organoids.

### 6.2 Application of 3D bioprinting technology in organoids

An emerging technique, 3D bioprinting technology involves fabricating *in vitro* 3D structural models using 3D printing technology, biological units, and biomaterials based on the functional requirements of living organisms (Liu et al., 2017). Traditional organoids are generated via stem cell expansion, specialization, and autonomous organization, lacking precise regulation over cell numbers, cell lineages, and the microenvironment (Mu et al., 2023). 3D bioprinting technology enables the construction of complex organoid structures through stable model building and multicellular-controlled organoid printing, allowing for the simultaneous printing of multiple cell components, ECM, and growth factors (Gong et al., 2021).

In 2020, Kim et al. (2020) proposed an organoid model based on 3D bioprinting technology, revealing the critical role of signaling crosstalk between tumor cells and stromal cells in controlling tumor plasticity. Chen et al. (2020) used 3D printing technology to fabricate a bio-scaffold and implant HCT116 human colon cancer cells, CAFs, and tumor-associated endothelial cells onto the scaffold, successfully constructing a 3D coculture colon cancer model. The 3D scaffold provided excellent support for the cells and helped maintain cell adhesion, proliferation, stemness, and vascularization. The activated stromal cells in the model exhibited high expression of various tumor-related factors and reshaped the ECM, while the tumor tissue showed transcriptomic characteristics highly similar to those *in vivo*.

Sbirkov et al. (2021) successfully constructed a novel 3D-printed model using Caco-2 human colon cancer cells. In this model, Caco-2 cells exhibited a realistic glandular-like histological morphology, and their RNA expression profile showed the upregulation of genes related to cell adhesion, hypoxia, and the EGFR/KRAS pathways and downregulation of genes related to cell cycle regulation. Kim and Kim (2020) described a novel bioprinting technique that uses cell-laden bio-ink composed of collagen and decellularized small intestine submucosa and successfully manufactured intestinal models with microvillus structures. These models exhibited physiological structures that closely resembled the intestine in terms of cell viability, alkaline phosphatase and aminopeptidase activity, permeability coefficient, and glucose uptake capacity.

### 6.3 Application of microfluidic devices in organoids

Microfluidic devices integrate sample preparation, reaction, separation, and detection into microchip platforms based on precision engineering, biomaterials, and tissue engineering. They enable the precise manipulation of tiny fluid flow through microchannels between different compartments and provide a technical platform for automated detection and analysis (Poenar, 2019). Microfluidic devices can be used to cultivate multiple cell types, organs, and tissues on the same platform, allowing for the precise control of component quantities, layouts, and spatial connections. When combined with techniques such as spheroids and organoids, microfluidic devices can construct complex and precise *in vitro* 3D models with high throughput, customization, low sample volume, and high efficiency (Saorin et al., 2023).

Microfluidic devices facilitate the reconstruction of complex TMEs and the simulation of microvascular systems, overcoming the limitations of traditional organoid models (Lin et al., 2017). Using a microfluidic device, Sontheimer-Phelps et al. (2020) successfully constructed a human colonic chip with a normal colonic mucus layer structure and function. Rajasekar et al. (2020) described a novel microfluidic system and co-cultivated self-assembled vascular networks with colonic organoids, achieving better organoid growth under constant perfusion conditions. Carvalho et al. (2019) incorporated HCT116 human colon cancer cells into a matrix gel as a tumor core and successfully constructed a CRC microfluidic chip with a vascular support network using human colonic microvascular endothelial cells. Shin et al. (2020) reported a patient-specific 3D physiodynamic mucosal chip that simulated *in vivo* intestinal fluid dynamics and cocultured it with the gut microbiome, resulting in the disease-specific differentiation of intestinal organoids. These data indicate that microfluidic devices hold significant promise and offer immense potential in tissue engineering, pharmaceutical research, and individualized healthcare.

## 7 Challenges and future directions

Organoids precisely replicate organ architecture and functionality, encompassing diverse cell types, tissue arrangements, and cellular interactions. These miniature organ models can be cultivated from a small number of cells or tissue samples and serve as effective tools for disease modeling and drug screening. They also hold potential for therapeutic interventions by enabling the reversal of pathogenetic mutations responsible for mutation-induced diseases (Carvalho et al., 2023). Using patients’ cells to construct organoids provides new possibilities for formulating personalized diagnosis and treatment programs (Betge and Jackstadt, 2023). Organoids have great potential in colorectal diseases. Using organoids for *in vitro* evaluation can influence clinical decision-making and improve the survival rate of patients. However, organoid technology is not yet fully mature and still faces many challenges.

The viability of organoid cultures is frequently hindered by the scarcity of viable cells in patient-derived specimens, posing a challenge to their successful establishment. Organoid culture techniques are still in the exploratory stages, with no consensus among experts regarding specific operational procedures (Li et al., 2020). Therefore, standardized protocols for organoid cultures are urgently needed. The high cost of growth factors and culture additives restricts the widespread adoption of organoid culture techniques. Moreover, adding multiple growth factors may induce genetic mutations, resulting in discrepancies between mechanistic exploration or drug sensitivity testing results and real-world scenarios (Lesavage et al., 2022). Despite their being valuable preclinical models for predicting drug efficacy, the full translation of organoids to clinical applications is challenging due to considerations such as drug side effects, underlying patient conditions, unconventional treatments, and medical ethics (Garreta et al., 2021).

Organoid technology offers immense potential for diverse applications across various fields. Organoids can be used to construct transplantable substitute organs, replacing diseased or damaged organs and providing temporary or permanent functional replacements, thereby improving patients’ quality of life (Rossi et al., 2018). Moreover, constructing PDO models can provide a deeper understanding of disease progression, assess the effectiveness of different treatment approaches, and establish a foundation for personalized medicine (Qu et al., 2021).

## 8 Conclusion

Organoids have the advantages of an appropriate culture cycle, stable passage, and high-throughput drug screening capability. They have broad application prospects in modeling CRC development and progression mechanisms and evaluating clinical treatment efficacy. While some shortcomings and limitations remain, new technological developments will produce a new generation of organoid models that even more faithfully recreate the *in vivo* picture. In summary, as preclinical models, organoids hold great potential to expedite the translation of basic research into clinical practice and provide valuable evidence to guide the clinical treatment of intestinal diseases.
